# Insights into the Association Between *Blastocystis* Infection and Colorectal Cancer

**DOI:** 10.1007/s11686-025-01079-y

**Published:** 2025-07-14

**Authors:** Amel Shehab, Mona El-Sayad, Amal Allam, Bassam Mohamed, Rasha Elsaka, Marwa Ibrahim, Naglaa Abd El-Latif

**Affiliations:** 1 Parasitology Department, Medical Research Institute, Alexandria University, Alexandria, Egypt; 2 Clinical Oncology and Nuclear Medicine, Faculty of Medicine, Alexandria University, Alexandria, Egypt; 3 Tropical Medicine Department, Faculty of Medicine, Alexandria University, Alexandria, Egypt

**Keywords:** *Blastocystis* spp., Colorectal cancer, PCR-RFLP analysis, PCR-STS analysis, ST distribution

## Abstract

**Objective:**

Given the unclear relationship between *Blastocystis* spp. infection and colorectal cancer (CRC), this study aimed to provide insight into *Blastocystis* infection, assess the effect of chemotherapy on Blastocystosis in CRC patients, and explore potential links between CRC and *Blastocystis* subtypes (STs).

**Subjects:**

A total of 150 participants were divided into three groups: Group I (50 CRC patients not receiving chemotherapy), Group II (50 CRC patients who were receiving chemotherapy), and Group III (50 healthy, age- and sex-matched controls).

**Results:**

*Blastocystis* spp. was detected in 42 cases through microscopy and culture, with infection rates higher in Group I (40%) and Group II (32%) compared to the control group (12%). Among all participants, 86 were asymptomatic, while 64 experienced symptoms. PCR analysis confirmed *Blastocystis* in 26 out of the 42 cases. PCR-restriction fragment length polymorphism (RFLP) analysis identified 60% of isolates as Group A and 16% as Group C, while sequencing later confirmed that 24% belonged to Group B. PCR-sequence-tagged site (STS) analysis revealed five STs (ST1, ST2, ST3, ST5, and ST7), with ST1 (52%) and ST3 (24%) identified as the most prevalent STs.

**Conclusion:**

*Blastocystis* infection was significantly higher in CRC patients, suggesting a possible association with the disease. It appears to act as an opportunistic pathogen, contributing to symptom development regardless of CRC. The absence of significant differences in ST distribution across groups indicates that *Blastocystis* pathogenicity is complex and not exclusively linked to specific STs.

## Introduction

*Blastocystis* spp. is a unicellular, anaerobic, eukaryotic protist commonly found in the intestinal tracts of humans and various animals. It is one of the most widespread intestinal microorganisms worldwide [1]. Infection rates range from 10% in developed countries to 60% in developing regions [2]. This organism exhibits diverse morphological forms, which contribute to its resilience and adaptability to varied environments. Its ability to transition between these forms may play a role in its persistence within the host and potentially influence its pathogenicity. Transmission occurs primarily through the fecal-oral route, with contaminated water and food being significant sources of infection [3].

In recent years, *Blastocystis* spp. has gained recognition as a protozoan potentially linked to gastrointestinal symptoms, though its pathogenicity remains controversial. It is frequently detected in both symptomatic and asymptomatic individuals, making its clinical significance unclear. While the exact mechanisms of pathogenicity are not fully understood, studies have associated infection with various non-specific symptoms, including abdominal pain, nausea, vomiting, anorexia, flatulence, and both acute and chronic diarrhea, as well as weight loss [4]. These symptoms are particularly pronounced in individuals with underlying health conditions, especially immunocompromised patients.

Chemotherapy-induced immunosuppression can increase susceptibility to opportunistic pathogens. Previous studies have reported higher *Blastocystis* infection rates in patients undergoing chemotherapy compared to healthy individuals [5]. This complex relationship emphasizes the need to examine its clinical impact on immunocompromised individuals and to evaluate how chemotherapy influences *Blastocystis* infection dynamics. A clearer understanding of this interaction is essential to manage *Blastocystis* infections in immunosuppressed patients.

Advancements in molecular diagnostics have significantly enhanced the detection and characterization of *Blastocystis* spp. Polymerase chain reaction (PCR)-based assays have become a widely used tool for identifying and subtyping *Blastocystis* from stool specimens with higher diagnostic accuracy than traditional methods. Restriction fragment length polymorphism (RFLP) analysis further aids in differentiating *Blastocystis* subtypes (STs) by digesting DNA at specific nucleotide sequences, generating unique fragment patterns that distinguish strains [6]. In addition, PCR-based sequence-tagged site (STS) analysis enables the identification and classification of distinct STs, improving the understanding of *Blastocystis* pathogenicity [7]. These molecular approaches provide crucial perception into *Blastocystis* infection patterns and their potential association with clinical outcomes.

Genetic analyses have identified significant diversity within *Blastocystis* spp., with at least 44 genetically distinct STs based on small subunit ribosomal RNA (SSU rRNA) gene sequences [8]. Of these, nine STs (ST1 to ST9) are known to colonize humans. This genetic variation has led to hypotheses that differences in pathogenicity and clinical symptoms among infected individuals may be linked to specific STs, potentially clarifying their role in symptomatic infections. ST3 exhibits the highest global distribution, followed closely by ST1 and ST2. However, the relationship between *Blastocystis* STs and disease remains inconclusive, warranting further molecular and clinical studies [9].

Colorectal cancer (CRC) is the third most diagnosed cancer worldwide and the fourth leading cause of cancer-related mortality [10]. Evidence suggests that various infectious agents including bacteria, viruses, and parasites may contribute to CRC risk by promoting cancer associated mechanisms. Recent studies propose that certain *Blastocystis* STs may increase CRC susceptibility, supporting tumor initiation and progression in specific populations [11, 12]. Some researchers hypothesize that *Blastocystis* may promote a pro-inflammatory environment in the gut, which could facilitate carcinogenesis through chronic immune activation and oxidative stress [13].

In view of the global burden of CRC and the emerging link between infections and cancer promoting pathways, this study aimed to provide insight into *Blastocystis* infection, examine the effect of chemotherapy on *Blastocystis* spp. infection in CRC patients, and explore whether specific *Blastocystis* STs are associated with the disease.

## Materials and methods

### Study Subjects

This study included 100 CRC patients attending the inpatient clinic of the Oncology Department, Alexandria Main University Hospital, Egypt, aged from 25 to 75 years. Females comprised the majority, accounting for 62% of the study population, while males represented 38%. Additionally, 50 healthy visiting relatives, with no history of cancer and matched for age and sex, agreed to participate as controls. Participants were categorized into three groups: Group I: 50 CRC patients not receiving chemotherapy, Group II: 50 CRC patients who were receiving chemotherapy and Group III: 50 healthy controls. Participants with intestinal co-infections were excluded from the study. Basic demographic and clinical data were collected from all participants using a predesigned questionnaire.

### Stool Sample Collection

Fresh stool samples were collected from every participant and transported to the Parasitology Department laboratory of the Medical Research Institute. Each sample was divided into three portions: one for direct microscopy, a second for culture, and a third for DNA extraction and PCR analysis.

### Microscopic Examination

Stool samples were thoroughly mixed and examined for *Blastocystis* spp. using saline and iodine wet mounts (Garcia, 2007) [14].

### In Vitro Culture Technique

*Blastocystis* spp. was cultured by inoculating ~ 50 mg of fresh stool into modified Jones’ medium, incubated at 37 °C, and the culture interface was examined at 24, 48, and 72 h (Jones, 1946) [15].

## Molecular Characterization of *Blastocystis* spp.

### DNA Extraction and PCR Amplification

DNA extraction was performed only on stool samples that tested positive for *Blastocystis*, using the ZYMO Fecal Isolate DNA Kit, following the manufacturer’s instructions. To enhance performance, β-mercaptoethanol was added to the stool DNA binding buffer at a final concentration of 0.5%. PCR amplification of the SSU rRNA gene was performed using the following primer set (Wong et al., 2008) [16]:

Blas-F: (5’-GGA GGT AGT GAC AAT AAA TC-3’)

Blas-R: (5’-ACT AGG AAT TCC TCG TTC ATG-3’)

The PCR reaction followed the procedure outlined by Mohamed et al. (2017) [17] and used Red Taq master mix (Bioline, UK). Each 25 µl reaction contained 12.5 µl of 2x Red Taq master mix, 1 µl each of 10 µM forward and reverse primers, 2 µl of template DNA, and 8.5 µl of nuclease-free water. PCR cycling conditions were as follows: an initial denaturation step at 95 °C for 4  min, followed by 35 cycles of denaturation at 95 °C for one minute, annealing at 50 °C for 30 s, and extension at 72 °C for one minute. Amplified products were visualized by UV transillumination after electrophoresis on a 1% agarose gel stained with ethidium bromide. Each run included a positive control (a previously sequenced PCR-positive sample) and a negative control (nuclease-free water).

### PCR-Restriction Fragment Length Polymorphism (RFLP) Analysis

PCR-RFLP analysis was used to group *Blastocystis* isolates, targeting a 1100 bp segment of the SSU rRNA gene. Amplified products were digested using the *SpeI* restriction enzyme (New England BioLabs, MA, USA), following the procedure described by Yoshikawa et al. (2011) [18].

### PCR- Sequence-Tagged Site (STS) Analysis

For subtyping, isolates were analyzed using PCR with seven sequence-tagged site (STS) primer pairs as described by Yoshikawa et al., (2004) [19] and Tan et al., (2009) [20]. Primer names, sequences, and expected product sizes are provided in Table 1. Amplifications were performed in 50 µl PCR reactions containing 5 µl of template DNA (10 ng/µl) and were carried out using an Applied Biosystems Veriti Thermal Cycler. PCR-RFLP products were separated by electrophoresis on a 2% agarose gel stained with ethidium bromide, and visualized under UV transillumination. Band sizes were compared to a 100 bp DNA ladder (Promega).

**Table 1:** Primer sets for differential identification of *Blastocystis* STs

STS primer sets	GenBank accession no.	Sequences	Product size	ST
SB83	AF166086	F-GAAGGACTCTCTGACGATGA R-GTCCAAATGAAAGGCAGC	351	1
SB155	AF166087	F-ATCAGCCTACAATCTCCTC R-ATCGCCACTTCTCCAAT	650	2
SB227	AF166088	F-ATCAGCCTACAATCTCCTC R-ATCGCCACTTCTCCAAT	526	3
SB332	AF166091	FGCATCCAGACTACTATCAACATT R-CCATTTTCAGACAACCACTTA	338	4
SB340	AY048752	F-TGTTCTTGTGTCTTCTCAGCTC R-TTCTTTCACACTCCCGTCAT	704	5
SB336	AY048751	F-GTGGGTAGAGGAAGGAAAACA R-AGAACAAGTCGATGAAGTGAGAT	317	6
SB337	AY048750	F-GTCTTTCCCTGTCTATTCTGCA R-AATTCGGTCTGCTTCTTCTG	487	7

### Sequencing of *Blastocystis* Isolates

To confirm genotypes identified by PCR-RFLP and to analyze samples that failed RFLP analysis, selected SSU rRNA gene-amplified products were sequenced. Sequencing was performed using the BigDye Terminator Cycle Sequencing Kit (Applied Biosystems) on an ABI Prism 310 Genetic Analyzer. Sequences were aligned using BioEdit version 7.0.1 and analyzed using BLAST program. Genotypes were confirmed by comparison with *Blastocystis* sequences in the NCBI database.

### Statistical Analysis

Data were analyzed using IBM SPSS software, version 20.0 (Armonk, NY: IBM Corp), with significance determined at a 5% level. Quantitative data were described in terms of number and percentage. The Chi-square test, with Monte Carlo correction, was employed to compare categorical variables across groups.

## Results

### Demographic Characteristics of the Studied Participants

Table 2 presents the age and gender distribution among the 150 study subjects. The majority of participants in all groups aged 25–<65 years, with a smaller proportion aged 65 and above. In terms of gender, females represented the majority in all groups, accounting for 62% of the total study population.

**Table 2:** Distribution of CRC patients and controls by age and gender

	Group I (*n* = 50)		Group II (*n* = 50)		Group III (*n* = 50)		Total (*n* = 150)	
	No.	%	No.	%	No.	%	No.	%
**Age (years)**								
25- < 45	26	52.0	22	44.0	20	40.0	68	45.3
45- < 65	20	40.0	18	36.0	17	34.0	55	36.7
> 65	4	8.0	10	20.0	13	26.0	27	18.0
**Gender**								
Male	17	34.0	19	38.0	21	42.0	57	38.0
Female	33	66.0	31	62.0	29	58.0	93	62.0

### Detection Rates of *Blastocystis* spp. Using Microscopy and Culture

Microscopically, *Blastocystis* spp. was detected in 40 cases (26.7%). The culture method identified *Blastocystis* spp. in 35 participants (23.3%), missing seven cases detected by microscopy but identifying two cases that were negative by microscopy (data not shown in the table). Thus the total number of infected participants was 42 (28%), the highest infection rate was in Group I (40%), followed by Group II (32%), with the lowest in Group III (12%). Statistical analysis showed significant differences between CRC patients and controls (Table 3).

**Table 3:** *Blastocystis* spp. detection among participants in the different groups using microscopy and culture

Groups	*Blastocystis* spp. negative		*Blastocystis* spp. positive		*p* value *x*^*2*^*=*10.317
	No.	%	No.	%	
Group I (50)	30	27.7	20	40	***p*** **=** 0.005* ***p1*** **=** 0.404 ***p2*** **=** 0.001* ***p3*** **=** 0.015*
Group II (50)	34	31.5	16	32	
Group III (50)	44	40.7	6	12	
Total (150)	108	72	42	28	

χ^2^: Chi square test

*p*: *p* value for comparing between the studied groups

*p*_*1*_: *p* value for comparing between Group I and Group II

*p*_*2*_: *p* value for comparing between Group I and Group III

*p*_*3*_: *p* value for comparing between Group II and Group III

*: Statistically significant at *p* ≤ 0.05

Group I: CRC patients not receiving chemotherapy

Group II: CRC patients on chemotherapy

Group III: Controls

### Age and Gender Characteristics of *Blastocystis* spp. Infected Cases

Table 4 shows that *Blastocystis* infection rates were slightly higher in younger and middle-aged adults (29.4% and 29.1%) compared to older participants (22.2%). Males had a significantly higher infection rate (38.6%) than females (21.5%).

**Table 4:** Distribution of *Blastocystis*-positive cases by age and gender

Demographic data	No. examined	No. positive	% positive	x^2^ *p* value
**Age (years)**				
25- < 45	68	20	29.4	
45- < 65	55	16	29.1	0.546 0.760
> 65	27	6	22.2	
All ages	150	42	28	
**Gender**				
Male	57	22	38.6	
Female	93	20	21.5	5.120 0.023*
Total	150	42	28	

*χ*^2^: Chi square test *: Statistically significant at *p* ≤ 0.05

*p: p* value for comparing between the studied groups

### **Clinical Data and Symptom Analysis among Participants**

Among the 150 participants 86 (57.3%) were asymptomatic, while 64 (43.3%) complained of various symptoms. Diarrhea and abdominal pain were the most commonly reported complaints; however, abdominal pain was the only symptom that showed a statistically significant difference. Nausea and vomiting were reported less frequently across all groups (Table 5).

**Table 5:** Clinical data among the 150 participants

Symptoms	Group I (*n* = 50)		Group II (*n* = 50)		Group III (*n* = 50)		Total (*n* = 150)		χ^2^	*P*
	No.	%	No.	%	No.	%	No.	%		
Asymptomatic	26	52.0	23	46.0	37	74.0	86	57.3	–	–
Symptomatic	24	48	27	54	13	26	64	43		
Nausea	2	4.0	3	6.0	0	0.0	5	3.3	2.878	^MC^*p*=0.371
Vomiting	2	4.0	1	2.0	1	2.0	4	2.6	0.680	^MC^*p*=1.000
Abdominal pain	9	18.0	15	30.0	1	2.0	25	16.6	14.208^*^	0.001^*^
Diarrhea	13	26.0	14	28.0	8	16.0	35	32.3	2.311	0.315

*x*^*2*^: Chi square test ^MC^*p*: Monte Carlo *: Statistically significant at *p* ≤ 0.05

*p: p* value for comparing between the studied groups

Group I: CRC patients not receiving chemotherapy

Group II: CRC patients on chemotherapy

Group III: Controls

Table 6 shows the association between *Blastocystis* infection and/or CRC regarding the absence or presence of symptoms. Participants were categorized into four groups: Group A: Controls/*Blastocystis-*negative (44/50), Group B: All CRC/*Blastocystis-*negative (64/100), Group C: All CRC/*Blastocystis-*positive (36/100) and Group D: All *Blastocystis-*positive (Controls and CRC) (42/150). No statistically significant difference was observed between *Blastocystis*-negative groups (A and B) and *Blastocystis*-positive groups (C and D).

**Table 6:** Association between *Blastocystis* infection, CRC and symptoms

Groups	No. examined	Asymptomatic		Symptomatic	
		No.	%	No.	%
A (Controls/*Blastocystis-*negative)	44/50	34	77	10	23
B (CRC/*Blastocystis-*negative)	64/100	49	76.5	15	23.5
C (CRC/*Blastocystis-*positive)	36/100	13	36	23	64
D (All *Blastocystis* positives)	42/150	16	38	26	62
*x* ^*2*^ *p* value		29.792 0.001*			
*p*1 *p*2		0.931 0.856			

*: Statistically significant at *p* ≤ 0.05

*x*^*2*^: Chi square test

### Impact of Chemotherapy on *Blastocystis* Infection Rates and Clinical Status in *Blastocystis-*infected CRC Patients

Table 7 shows that *Blastocystis* infection rates were higher in Group I (40%) than in Group II (32%), with no significant difference between the groups. It also compares the clinical condition of *Blastocystis*-infected CRC patients. In Group I, 45% were asymptomatic and 55% symptomatic, whereas Group II had fewer asymptomatic cases (25%) and more symptomatic cases (75%).

**Table 7:** Effect of chemotherapy on *Blastocystis* infection and on the presence or absence of symptoms in *Blastocystis*-infected CRC patients

Groups	Blastocystosis							
	Infection rate				Asymptomatic		Symptomatic	
	No. +ve	% +ve	No. -ve	% -ve	No. +ve	% +ve	No. -ve	% -ve
Group I (50)	20	40	30	60	9	45	11	55
Group II (50)	16	32	34	64	4	25	12	75
*x* ^*2*^ *p* value	0.694 0.404				1.541 0.214			

*χ*^2^: Chi square test

*p: p* value for comparing between the studied groups

Group I: CRC patients not receiving chemotherapy

Group II: CRC patients on chemotherapy

### Results of PCR among Positive Cases

**Fig. 1:**
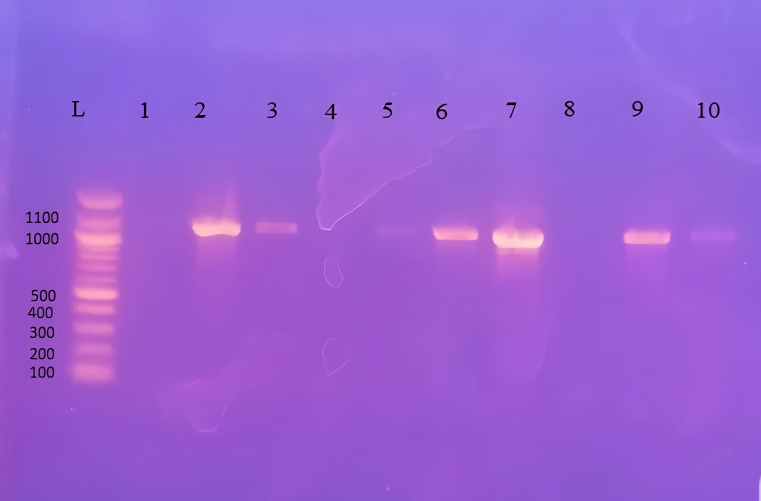
Demonstrative 1% agarose gel with amplification of the *Blastocystis*-specific 1100 bp target of the SSU rRNA gene. Lane L contains the 100 bp DNA ladder; Lane 1 is the negative control; Lanes 2, 3, 6, 7, 9 and 10 show positive results for *Blastocystis* spp.; and Lanes 4, 5, and 8 show negative results for the *Blastocystis* spp. gene

As shown in Table 8; Fig. 1, molecular analysis was performed on 42 *Blastocystis*-positive stool samples. DNA extraction confirmed 26 positive cases, including 12 from Group I, 10 from Group II, and 4 from Group III.

**Table 8:** *Blastocystis* spp. among positive cases using PCR

PCR	Group I (*n* = 20)		Group II (*n* = 16)		Group III (*n* = 6)		Total (*n* = 42)	
	No.	%	No.	%	No.	%	No.	%
Positive	12	60	10	62.5	4	66.6	26	61.9
Negative	8	40	6	37.5	2	33.4	16	38.1

Group I: CRC patients not receiving chemotherapy

Group II: CRC patients on chemotherapy

Group III: Controls

### Grouping and Subtyping of *Blastocystis* by PCR-RFLP and STS-PCR Analysis

**Fig. 2:**
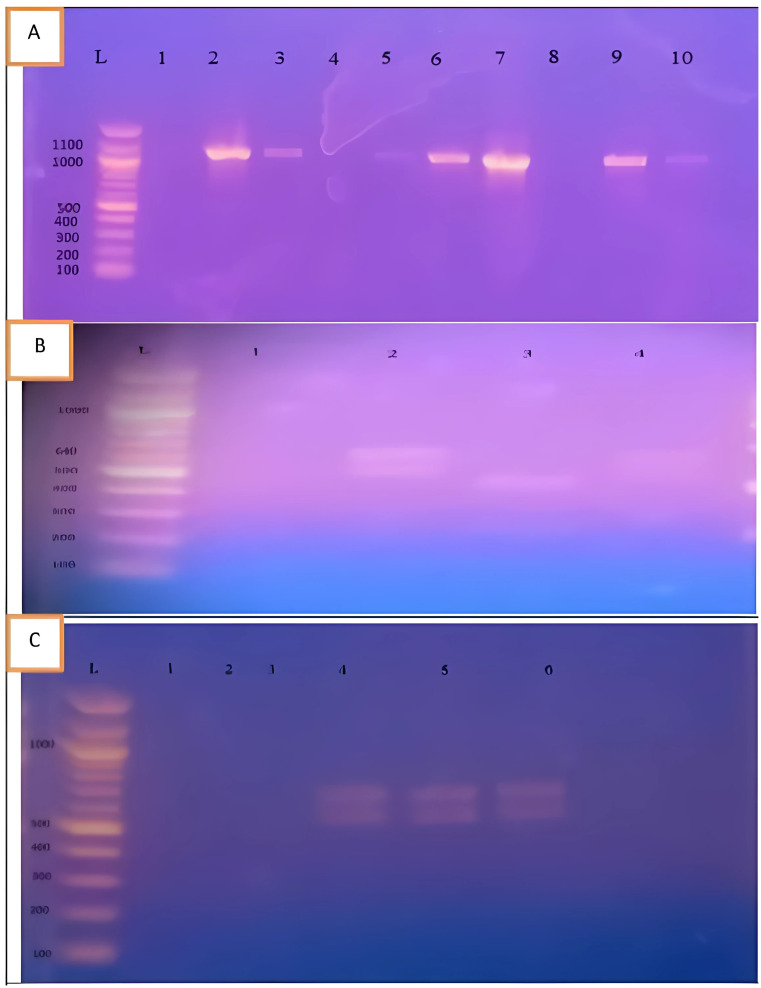
RFLP and STS-PCR analysis of *Blastocystis* spp. **A**) RFLP-PCR of the 1100 bp SSU rRNA gene showing digestion patterns for *Blastocystis* Group A (≈ 450 bp and 200 bp, Lane 3) and Group C (≈ 470 bp and 640 bp, Lanes 2 and 4); Lane L: 100 bp DNA ladder. **B**) RFLP-PCR profile for Group C (Lanes 3–5); Lane L: 1000 bp DNA ladder. **C**) STS-PCR showing STs: ST1 (400 bp, Lane 1) and ST5 (700 bp, Lanes 2 and 6); Lane L: 100 bp DNA ladder

*Blastocystis* isolates were grouped based on RFLP analysis of the amplified 1100 bp SSU rRNA gene target. Two groups were identified: Group A (≈ 200 bp and 450 bp) with 15 isolates and Group C (≈ 470 bp and 650 bp) with 4 isolates (Fig. 2). PCR-RFLP analysis did not identify Group B, which was later detected through sequencing. PCR-STS analysis further subtyped Group A into ST1 (52%) and ST2 (8%) and Group C into ST5 (12%) and ST7 (4%).

### DNA Sequencing

To confirm the genetic classification, 13 samples (seven RFLP-failed cases, three weak band cases, and three randomly selected positive samples) were further analyzed by SSU rRNA gene sequencing. BLAST analysis identified six isolates as Group B, representing 24% of the total detected isolates. Results of the sequencing data are summarized in Table 9.

**Table 9:** Genotyping results of 13 sequenced samples with GenBank accession numbers

Subtypes	Patients group	GenBank accession number
ST1	I	KY823345.1
ST1	I	MK375239.1
ST1	II	KY823345.1
ST2	I	MK801368.1
ST3	I	MT330277.1
ST3	II	MC330277.1
ST3	II	MN914081.1
ST3	II	MK375226.1
ST3	III	MK375277.1
ST3	III	MK375225.1
ST5	I	KC148209.1
ST5	II	EF209016.1
ST7	I	MT898459.1

Group I: CRC patients not receiving chemotherapy

Group II: CRC patients on chemotherapy

Group III: Controls

### Frequency of *Blastocystis* spp. STs

ST1 was the most prevalent *Blastocystis* ST, detected in 50% of Group I and 66.7% of Group II. ST3 was the second most common, while ST5 and ST7 were less frequent, with ST7 found only in Group I. In Group III, ST3 was the most common (50%), followed by ST1 and ST2 (25% each). No statistically significant differences were observed in ST distribution among the groups (Table 10).

**Table 10:** Distribution of *Blastocystis* STs among the study groups

Subtypes	Group I no. Infected (12)	Group II no. Infected (9)	Group III no. Infected (4)	Total STs (25)	χ^2^	^MC^ *p*
ST1	6 (50.0%)	6 (66.7%)	1 (25.0%)	13 (52%)	1.887	0.435
ST2	1 (8.3%)	0 (0.0%)	1 (25.0%)	2 (8%)	2.358	0.423
ST3	2 (16.7%)	2 (22.2%)	2 (50.0%)	6 (24%)	1.917	0.391
ST5	2 (16.7%)	1 (11.1%)	0 (0.0%)	3 (12%)	0.678	1.000
ST7	1 (8.3%)	0 (0.0%)	0 (0.0%)	1 (4%)	1.462	1.000
χ^2^ (π)	**6.493 (0.718)**					

**χ**^**2**^: Chi square test ^**MC**^***p***: Monte Carlo

Group I: CRC patients not receiving chemotherapy

Group II: CRC patients on chemotherapy

Group III: Controls

## Discussion

A growing body of research suggests that *Blastocystis* spp. may play a role in CRC development. In the present study, among the 150 participants, *Blastocystis* spp. was detected in 42 cases (28%) using both microscopy and culture. Microscopy identified *Blastocystis* in 26.7% of cases with 100% specificity, attributed to the expertise of highly skilled technicians. However, culture, often considered the gold standard, showed a lower detection rate (23.3%). This discrepancy may be due to challenges in the culture process, resulting from microbial interference or parasite degradation during incubation. The interplay between patient health status and detection methodologies appears to be a key factor influencing *Blastocystis* spp. prevalence in CRC patients. A higher infection rate was observed in CRC patients compared to healthy controls. Consistently, Sulżyc-Bielicka et al. (2021) [21] reported a higher *Blastocystis* prevalence in CRC patients (12.15%) compared to controls (2.42%) using light microscopy. In contrast, Ali et al. (2021) [22] found higher prevalence rates in both CRC patients (52%) and controls (42%) using in vitro culture. These inconsistencies suggest the necessity for employing multiple diagnostic methods to achieve more accurate estimates of *Blastocystis* prevalence.

*Blastocystis* infection rates did not significantly differ across age groups. However, a significantly higher infection rate was observed in males compared to females (38.6% vs. 21.5%).

The pathogenicity of *Blastocystis* spp. remains a subject of debate, as evidenced by its detection in both symptomatic and asymptomatic individuals. In this study, 86 out of 150 participants (57%) were asymptomatic, while 64 (43%) reported symptoms. Abdominal pain and diarrhea were the most prevalent, while other symptoms were less frequent and showed no significant variation across groups. Other studies, including one by Abu Sheishaa et al. (2023) [23], reported a higher prevalence of *Blastocystis* in asymptomatic individuals, suggesting weak association with clinical symptoms. Similarly, a study conducted in Senegal found that symptomatic *Blastocystis*-infected patients frequently experienced diarrhea, abdominal pain, and dyspeptic disorders, although many asymptomatic carriers were also identified [24].

Regarding *Blastocystis* infection and CRC, in the present study, *Blastocystis* infection rates were higher in CRC patients (irrespective of treatment) compared to controls. Previous studies have reported higher *Blastocystis* infection rates in patients undergoing chemotherapy compared to healthy individuals. A 2023 case-control study in the UAE found *Blastocystis* in 40.4% of cancer patients versus 17.3% of healthy controls [11]. Likewise, a 2021 study in Poland found *Blastocystis* infection to be five times more likely in CRC patients (12.6%) than in controls (2.6%) [21]. These findings suggest that the immunocompromised status associated with cancer may contribute to increased *Blastocystis* spp. prevalence.

The inclusion of both chemotherapy and non-chemotherapy CRC patients aimed to assess whether chemotherapy influences *Blastocystis* infection rate. Chemotherapy is known to alter the gut microenvironment, potentially affecting parasite survival and reducing *Blastocystis* spp. viability. While it may exert a toxic effect on the parasite, ultimately limiting its survival, *Blastocystis* spp. has the ability to modulate the host immune response which may contribute to its persistence despite chemotherapy. The parasite employs several mechanisms to evade immune responses, including the degradation of secretory immunoglobulin A (sIgA), evasion of host defenses, and alteration of cytokine production, such as interleukin-8, which disrupts local immune responses [25]. These adaptations enable *Blastocystis* spp. to persist in immunocompromised hosts, where a weakened immune system may struggle to control colonization. This dual effect, where chemotherapy-induced immunosuppression may decrease parasite viability, yet *Blastocystis*’ immune-modulating properties allow it to persist, creates a complex interaction that calls for further study [26].

Regarding the impact of chemotherapy on the clinical condition of *Blastocystis*-infected CRC patients, a lower proportion of symptomatic cases was observed in Group I (55%) compared to Group II (75%), despite the lower infection rate in chemotherapy-treated patients. Although this difference did not reach statistical significance (*p* = 0.214), this suggests that while chemotherapy may lower infection rates it does not necessarily alleviate symptoms in *Blastocystis*-infected CRC patients and could even contribute to increased symptom manifestation, possibly due to chemotherapy-induced immunosuppression.

A comparative analysis was conducted to determine whether *Blastocystis* infection or CRC plays a more significant role in symptom development. The comparison between *Blastocystis*-negative controls (Group A) and *Blastocystis*-negative CRC patients (Group B) revealed no significant difference, indicating that CRC alone does not substantially contribute to gastrointestinal symptoms in the absence of *Blastocystis* infection. Similarly, the comparison between *Blastocystis*-positive CRC patients (Group C) and all *Blastocystis*-positive individuals (Group D) showed no statistically significant difference (*p*2 = 0.856). These findings reinforce the idea that *Blastocystis* infection, rather than CRC itself, plays a primary role in symptom development, supporting its classification as a potential opportunistic pathogen. In line with these findings, Stensvold et al. (2009) [27] suggested a possible association between *Blastocystis* infection and CRC development, potentially exacerbating gastrointestinal symptoms. Likewise, Mohamed et al. (2017) [17] reported a significant presence of *Blastocystis* in CRC patients but found no direct correlation with specific gastrointestinal manifestations.

In the present study, PCR analysis detected *Blastocystis* spp. in 26 out of 42 (61.9%) of positive samples. This detection rate is low compared to traditional methods like microscopy and culture. Factors such as DNA degradation, ineffective extraction, or the presence of inhibitors in stool samples may contribute to this reduced detection rate, influencing detection outcomes. These challenges align with findings from other studies, where PCR detection rates varied based on sample quality, the choice of PCR primers and protocols can significantly impact detection sensitivity, as variations in target gene regions and amplification conditions may lead to inconsistent results across different studies [28, 29].

Conversely, other studies have reported higher detection rates of *Blastocystis* in CRC patients using PCR. A study conducted in Poland found that PCR detected *Blastocystis* in 12.63% of CRC patients, which was significantly higher than in the control group (2.63%) [20]. Similarly, research from the UAE reported a 60% prevalence of *Blastocystis* in CRC patients using molecular methods [11].

PCR-RFLP analysis revealed that most *Blastocystis* isolates belonged to Group A (60%), and Group C (16%). Further subtyping with PCR-STS primers identified five distinct STs (ST1, ST2, ST3, ST5, and ST7), with ST1 (52%) and ST3 (24%) being the most predominant. ST1 was most frequent among non-chemotherapy CRC patients, a pattern potentially linked to its association with gastrointestinal symptoms and potential pathogenicity. However, no significant differences in ST distribution were observed between CRC patients and healthy controls, suggesting that *Blastocystis* infection itself, rather than a specific ST, may be associated with CRC development. The relationship between *Blastocystis* STs and CRC remains complex, as studies present conflicting evidence [21, 30, 31].

While some studies suggest a potential pathogenic role for specific STs [12, 17], others [30, 31], including the present study, found no significant differences in ST distribution between CRC patients and healthy controls. This inconsistency highlights the intricate nature of *Blastocystis* involvement in CRC. Several studies have investigated this association further, Khaled et al. [32] and Kumarasamy et al. [12] reported frequent detection of *Blastocystis* in CRC patients, with ST1 and ST3 being the most common STs. They proposed that *Blastocystis* may contribute to CRC progression by altering the host immune response and increasing oxidative stress. Conversely, Mohamed et al. (2017) [17] identified a significant association between ST1 and CRC, suggesting a ST-specific role in carcinogenesis. They hypothesized that ST1 might influence CRC development by inhibiting apoptosis in colon cancer cells and promoting their proliferation through the downregulation of host immune responses [33].

This study identified only single-subtype infections. The absence of mixed ST infections could be attributed to several factors. One possible explanation is the selective outgrowth of a dominant ST, where certain STs may have a competitive advantage within the host environment, leading to their predominance. Additionally, competitive interactions between STs within the host may influence which ST prevails, potentially limiting the coexistence of multiple STs. This suggests that the pathogenic potential of *Blastocystis* may be more closely linked to factors such as the host’s immune response, specific ST characteristics, and interactions with the intestinal microbiota. In contrast, mixed infections have been reported in other populations [34]. A study conducted in Colombia identified mixed infections in 8.7% of samples, with ST1 and ST3 being the most prevalent combinations [34].

In conclusion, this study suggests a potential link between *Blastocystis *spp. infection and CRC, though no significant differences in ST distribution were observed. *Blastocystis* may act as an opportunistic pathogen contributing to gastrointestinal symptoms, while chemotherapy appears to reduce infection rates without easing symptoms, possibly due to immune suppression. Further research is needed to determine whether *Blastocystis* is a true risk factor for CRC or merely an opportunistic colonizer in immunocompromised patients.

Limitations of the molecular study include the relatively small sample size, which may affect the precision and reliability of the results.

## Data Availability

All data generated or analyzed during this study are included in this published article.
